# Nucleotide Polymorphisms and Haplotype Diversity of *RTCS* Gene in China Elite Maize Inbred Lines

**DOI:** 10.1371/journal.pone.0056495

**Published:** 2013-02-20

**Authors:** Enying Zhang, Zefeng Yang, Yifan Wang, Yunyun Hu, Xiyun Song, Chenwu Xu

**Affiliations:** 1 Key Laboratory of Crop Genetics and Physiology of Jiangsu Province, Key Laboratory of Plant Functional Genomics of the Ministry of Education, College of Agriculture, Yangzhou University, Yangzhou, China; 2 College of Agronomy and Plant Protection, Qingdao Agricultural University, Qingdao, China; San Francisco Coordinating Center, United States of America

## Abstract

The maize *RTCS* gene, encoding a LOB domain transcription factor, plays important roles in the initiation of embryonic seminal and postembryonic shoot-borne root. In this study, the genomic sequences of this gene in 73 China elite inbred lines, including 63 lines from 5 temperate heteroric groups and 10 tropic germplasms, were obtained, and the nucleotide polymorphisms and haplotype diversity were detected. A total of 63 sequence variants, including 44 SNPs and 19 indels, were identified at this locus, and most of them were found to be located in the regions of UTR and intron. The coding region of this gene in all tested inbred lines carried 14 haplotypes, which encoding 7 deferring RTCS proteins. Analysis of the polymorphism sites revealed that at least 6 recombination events have occurred. Among all 6 groups tested, only the P heterotic group had a much lower nucleotide diversity than the whole set, and selection analysis also revealed that only this group was under strong negative selection. However, the set of Huangzaosi and its derived lines possessed a higher nucleotide diversity than the whole set, and no selection signal were identified.

## Introduction

In the past, fundamental researches on increasing shoot biomass and seed yield attracted most attentions of the crop scientists, and the relevance of the root system for food production has often been overlooked [1], [2]. However, a healthy and well-developed root stock architecture is especially important for the developing of plant, because it is the organ absorbing water and inorganic nutrients, in addition to anchoring of the plant body to the ground [1], [3]. Maize (*Zea mays* L.), one of the most widely grown grain crop in the world, possesses a unique and complex root stock architecture composed of embryonic and postembryonic roots [4], [5]. The embryonic roots, defined by the primary root and a variable number of seminal roots, play important roles for early vigor of the maize seedlings. However, at the postembryonic stage, shoot-borne system forms the major backbone of the adult stock [6].

Recently, several genes controlling the development of maize shoot-borne roots, lateral roots, and root hairs have been isolated [7], [8], [9]. Among them, the gene *RTCS* (rootless concerning crown and seminal roots) was demonstrated to play a central role in the auxin-mediated initiation of seminal and shoot-borne roots in maize [5], [9] and the mutant of this gene was impaired in the formation of these roots. Map-based cloning revealed that this gene was located in the short arm of chromosome 1, and encoded a LOB domain protein. Sequence analysis illustrated the maize *RTCS* gene was composed of 2 exons, separated by a 96-bp intron, and its protein product contained 244 amino acid residues. The maize *RTCS* gene is preferentially expressed in root tissues [5] and its protein product showed typical features of a transcription factor including nuclear localization, DNA-binding and downstream gene activation [6].

Although the favorable root architecture plays critically important roles for the development of plant, root architecture was rarely considered as a selection criterion or traits for maize improvement, mainly because of the practical difficulties with their evaluation under field conditions [3]. Recent researches in maize revealed that changes in root architecture can strongly affect the yield [10]. Because increasing crop yield through improvement of plant type and growing use of fertilizer has reached a maximum, much attention should be focused on improving the root system [1]. Researches on the sequence polymorphisms of key genes are important not only for crop improvement but also for efficient management and conservation of plant genetic resources [11], [12], [13]. However, rare researches in genetic variants in the DNA sequence have focused on the genes controlling the development of plant roots. In addition, the genetic diversity at the DNA level of maize *RTCS* gene is not known at present. Therefore, we detected nucleotide polymorphisms, haplotype diversity and evolutionary factors of the gene *RTCS* by direct sequencing 73 China elite inbred lines, including the lines from 5 temperate heterotic groups and some tropic germplasms.

## Materials and Methods

### Plant Materials

A total of 73 China maize elite inbred lines were used in this study (Table 1). Among these inbred lines, 63 temperate and 10 tropic germplasms were used. The 63 temperate inbred lines were from 5 heterotic groups, including 15 from Tangsipingtou, 9 from Lvdahonggu, 11 from Lancaster, 13 from Reid, and 14 from P group.

**Table 1:** List of the 73 inbred lines included in this study.

No.	Inbred line	Heterotic group	No.	Inbred line	Heterotic group	No.	Inbred line	Heterotic group
1	QH19612^a^	Tangsipingtou	26	4CV	Lancaster	50	178	P group
2	Chang7-2^a^	Tangsipingtou	27	Qi232	Lancaster	51	QP1721	P group
3	LX9801^a^	Tangsipingtou	28	OH43	Lancaster	52	Exhan	P group
4	107	Tangsipingtou	29	MO17	Lancaster	53	xy35	P group
5	Huang518^a^	Tangsipingtou	30	BJ-4	Lancaster	54	P138	P group
6	k12^a^	Tangsipingtou	31	BEM	Lancaster	55	6819	P group
7	H21^a^	Tangsipingtou	32	BJ-1	Lancaster	56	Dan988	P group
8	Ji853^a^	Tangsipingtou	33	BJ-3	Lancaster	57	319B	P group
9	Za107	Tangsipingtou	34	BJ-5	Lancaster	58	Qi319	P group
10	Huangzaosi^a^	Tangsipingtou	35	412	Lancaster	59	Qi318	P group
11	502^a^	Tangsipingtou	36	8112	Reid	60	Shen137	P group
12	Luyuan92	Tangsipingtou	37	K8112	Reid	61	91158	P group
13	10168	Tangsipingtou	38	Wu314^a^	Reid	62	s80	P group
14	QZ01^a^	Tangsipingtou	39	4866	Reid	63	Danhuang25	P group
15	Y53	Tangsipingtou	40	3189	Reid	64	11099	Tropic
16	Dan598	Lvdahonggu	41	Tie9206	Reid	65	suwan	Tropic
17	Zong3	Lvdahonggu	42	Benyu15	Reid	66	11118	Tropic
18	E28	Lvdahonggu	43	Chun2433	Reid	67	11200	Tropic
19	Dan340	Lvdahonggu	44	478Xuan	Reid	68	10533-1	Tropic
20	Zi330	Lvdahonggu	45	Zheng58	Reid	69	GB28	Tropic
21	S122	Lvdahonggu	46	7922	Reid	70	RCML15	Tropic
22	340Gai	Lvdahonggu	47	8605-2	Reid	71	DK3110	Tropic
23	JH3372	Lvdahonggu	48	JB	Reid	72	RBS11	Tropic
24	Dan99	Lvdahonggu	49	B73	Reid	73	FLB01	Tropic
25	nx335	Lancaster						

a The inbred lines of Huangzaosi and its derived lines.

### DNA-extraction and Sequencing *RTCS* Gene

Genomic DNA of was extracted from young leaves of the tested inbred lines at the seedling stage using CTAB (cetyl trimethyl ammonium bromide) method based on the modified protocol [14]. The sequences of the *RTCS* gene in 73 inbred lines was sequenced by BGI Life Tech Co., Ltd. using the target sequence capture sequencing technology on the NimbleGen platform [15].

### Sequence Analysis

Multiple sequence alignment was performed using Clustal X [16] and was further edited manually. The software DNASP 5.0 [17], [18] was used to analyze sequence nucleotide polymorphism and allelic diversities. Two parameters of nucleotide diversity, 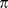 and 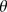 were estimated. Where 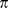 is the average number of nucleotide differences per site between any two DNA sequences, and 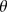 is derived from the total number of segregating sites and corrected for sampling size. Tajima’s D [19] and Fu and Li’s [20] statistical tests were used to test the evidence of neutral evolution within each group and each defined region. The minimum number of recombination events [21] was estimated in the period of evolution of *RTCS* gene among these inbred lines.

## Results

### Nucleotide Diversity and Selection of *RTCS* Gene in China Elite Inbred Lines

**Figure 1:**
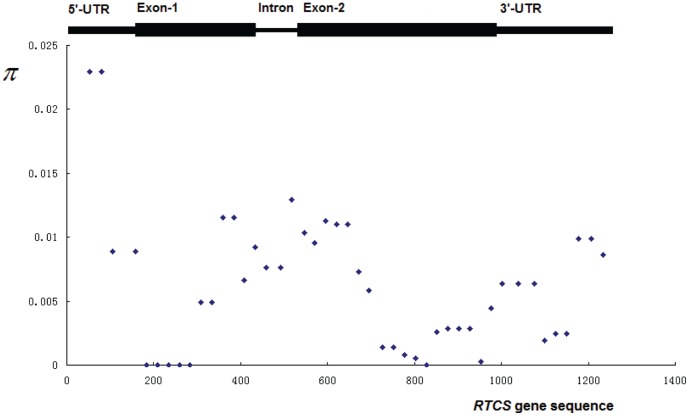
The nucleotide diversity (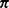) estimated along the *RTCS* gene sequences. 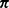
 is calculated in sliding windows of 100 bp using a step size of 25 bp. 5 regions of *RTCS* gene, including 5′ UTR, Exon-1, Intron, Exon-2, and 3′ UTR, were indexed on the top of the coordinate.

Sequence polymorphisms were detected among 73 maize inbred lines across 1279 bp of sequence, which covers a 167 bp 5′ untranslated region (UTR), a 735 bp coding region, a 104 bp intron region, and a 273 bp 3′ UTR. Nucleotide substitutions and indels at the *RTCS* locus were identified, and the results were summarized in Table 2. From the putative genomic sequences of the 73 maize inbred lines, a total of 44 SNP sites were identified, and among them, 16 and 28 sites belonged to singleton variable sites and parsimony informative sites, respectively. In addition, a total of 19 indel events covering 90 sites were identified in the genomic sequences (Table S1). For all the 73 inbred lines, the overall nucleotide diversity (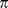) of *RTCS* locus was 0.00666. Among 4 regions of the gene *RTCS*, the coding region showed much lower nucleotide polymorphism than others, while the intron region had the highest frequency of all sequence variants. This might be caused by the variant of indels, because this region had the highest frequency of indels per bp. However, the frequency of nucleotide substitutions in 5′UTR was higher than other regions. When we used the sliding window of 100 bp under a step size of 25 bp, the result revealed each region of the *RTCS* sequence possessed high frequency of polymorphic sites (Figure 1). The highest nucleotide diversity was within 1–159 bp in 5′-UTR with 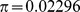, while the lowest value (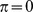) was found in regions of exon-1 and exon-2, respectively. The observed distribution of SNP sites and indel sites was found to be significantly different (for SNP, 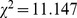, 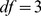, 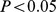; for indel, 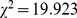, 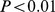) from an expected even distribution across the four defined regions (Table 3). The uneven distribution of polymorphisms might be particularly due to the low frequency of variants in coding region.

**Table 2:** Summary of the frequency of polymorphisms.

Parameters	5′UTR	Coding region	Intron	3′UTR	Entire region
Total length of amplicons (bp)	167	735	104	273	1279
Number of all sequence variants (SNPs and indels)	16	16	11	20	63
Frequency of all sequence variants	0.0958	0.0218	0.1058	0.0733	0.0493
Number of nucleotide substitutions (bp)	10	14	6	14	44
Frequency of polymorphic sites per bp	0.0599	0.0190	0.0577	0.0513	0.0344
Number of indels	6	2	5	6	19
Number of indel sites	34	6	12	38	90
Average indel length	5.6667	3	2.4	6.3333	4.7368
Frequency of indels per bp	0.0359	0.0027	0.0481	0.0220	0.0146

**Table 3:** Nucleotide and allele diversities of *RTCS* gene by analyzing 73 maize inbred lines.

Parameters	5′UTR	Codingregion	Intron	3′UTR	Entireregion
*π*	0.01726	0.00436	0.00860	0.00702	0.00666
*θ*	0.01547	0.00395	0.01342	0.01226	0.00761
Tajima’ D	0.31120	0.29566	−0.85026	−1.22021	−0.40940
Fu and Li’s D*	1.38655	−1.63477	−2.51473*	−1.63477	−1.52039
Fu and Li’s F*	1.20720	−1.13533	−2.32168*	−1.76828	−1.30730

* indicates the significance at *P*<0.05 level.

The Tajima’s D test is a widely used test to identify sequences which do not fit the neutral theory model at equilibrium between mutation and genetic drift [19]. All the values of Tajima’s D in the present study were not statistically significant, illustrating no significant selection existed in the entire *RTCS* sequences. In addition, Fu and Li’s D* and F* were also not significant in almost all regions except for intron. Although these results could not reject the hypothesis of mutation drift equilibrium, a lack of footprint of positive selection in most regions of *RTCS* was suggested.

### Nucleotide Diversity and Selection in Each Heterotic Group

The inbred lines used in this study can be classified into 6 groups, including 5 temperate heterotic groups and the group of tropic germplasms. We also tested the nucleotide diversity of both entire region and coding region of *RTCS* sequences for each group, and the result revealed that the nucleotide diversities of 5 groups were higher than or very near to the whole set (Table 4). The tropic group possessed the highest value nucleotide diversity, and its haplotype diversity (*Hd*) is 1 for the entire region of *RTCS*, suggesting each inbred line carried a haplotype. Only the P heterotic group had much lower nucleotide and haplotype diversities than the whole set both for the entire region and coding region. This result suggested that the P group was more conserved in *RTCS* locus than other groups. In addition, we also noticed that the statistics for Tajima’s D, Fu and Li’s D* and F* were all statistically significant in P group. This result illustrated that the *RTCS* gene in P group were not evolved neutrally, and also suggesting that selection might only acted the evolution of *RTCS* gene in this group.

**Table 4:** Nucleotide and allele diversities of *RTCS* gene in each heterotic group.

Parameters	Tangsipingtou		Lvdahonggu		Lancaster		Reid		P group		Tropic lines		Huangzaosi and its derived lines	
	Entire region	CDS	Entire region	CDS	Entire region	CDS	Entire region	CDS	Entire region	CDS	Entire region	CDS	Entire region	CDS
*π*	0.00941	0.00484	0.0064	0.00402	0.00746	0.00392	0.00706	0.00413	0.00081	0.00078	0.01155	0.00661	0.00737	0.00447
*θ*	0.00828	0.00336	0.00659	0.00302	0.00858	0.00373	0.00768	0.00352	0.00178	0.00172	0.01219	0.00679	0.00806	0.00373
*Hd*	0.9714	0.8476	0.8889	0.7500	0.8727	0.7091	0.978	0.8718	0.2747	0.1429	1	0.9333	0.9818	0.8182
Tajima’ D	0.57302	1.62023	−0.14261	1.45615	−0.60687	0.21418	−0.34215	0.67257	−2.01359*	−1.79759*	−0.25538	−0.11714	−0.3956	0.82009
Fu and Li’s D*	0.5794	1.36102*	−0.32781	1.37224	−1.28402	−0.46234	−0.7663	0.88217	−2.60025*	−2.2738*	−0.14763	−0.41245	−0.74657	0.46649
Fu and Li’s F*	0.66505	1.63999*	−0.31725	1.55087	−1.25975	−0.32956	−0.7467	0.94243	−2.79240*	−2.44883*	−0.19709	−0.38199	−0.74484	0.62774

* indicates the significance at *P*<0.05 level.

Huangzaosi is believed to be the representative line of the Tangsipingtou heterotic group and was used as a key maize inbred line in China [22]. Among all inbred lines used in this study, at least 11 lines were Huangzaosi and its derived lines. We also tested the sequence polymorphisms of *RTCS* gene in Huangzaosi and its derived lines. The result revealed that the nucleotide diversity (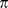) is higher than the whole set, illustrating that there were abundant nucleotide variations in Huangzaosi and its derived lines. In addition, none of the statistics for Tajima’s D, Fu and Li’s D* and F* were statistically significant for Huangzaosi and its derived lines, suggesting that selection was not included in the *RTCS* locus of this population.

### Haplotype Diversity

Based on the whole length of the *RTCS* gene sequenced in 73 maize inbred lines, a total of 34 haplotypes were detected with a *Hd* equal to 0.8992 (Table S2). The inbred lines were unbalancedly distributed in these haplotypes. Among the haplotypes identified in this analysis, 26 contained only one inbred line. The most frequent haplotype was Hap_8, which contained 21 inbred lines. It should be mentioned that nearly all the inbred lines in P group belonged to this haplotype except for 91158, which was assigned to the haplotype Hap_27.

In the coding region of the gene *RTCS*, 16 sequence variants, including 2 indels and 14 SNPs, were detected. Both of the 2 indels contained 3 nucleotide acids, respectively, and this can not result in frameshift of the codons. When we used the coding sequences to identify the hapotype diversity, a total of 14 haplotypes were identified for these 73 inbred lines (Table S3), and the hapotype diversity was 0.7705. Among the haplotypes identified according to CDS, 9 contained only one inbred line. The most frequent CDS haplotype was CDS_Hap_5, which contained 29 inbred lines from all 6 groups. In addition, CDS_Hap_7 and CDS_Hap_8 were also haplotypes with high frequency, and only no inbred lines in P group and tropic lines carried them, respectively.

**Figure 2:**
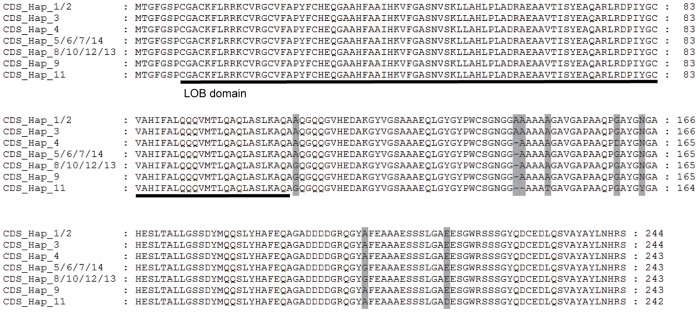
Sequence alignment of RTCS proteins encoded by different CDS haplotypes. The hyplotypes identified by coding sequences of *RTCS* gene were used as sequence name. Polymorphisms from inferred amino acid were indicated by shade.

Among 14 SNPs in the coding region, 8 were synonymous sites, and the other 6 were nonsynonymous sites. The nonsynonymous sites and the indels will lead to the changes of protein sequences. When we translated the CDS into amino acid sequences, 7 types of RTCS protein sequences were found to be encoded by these inbred lines (Figure 2). Haplotypes CDS_Hap_5/6/7/14 encoded the most frequent type of RTCS protein, and contributed to more than half of all the inbred lines (42 out of 73). The variation of RTCS protein sequences was the result of combinations of 6 nonsynonymous mutations and 2 indels in the coding region. All of the variants at the protein level were found to be outside the LOB domain region (Figure 2), and in other words, the region of LOB domain of RTCS protein showed 100% identify in all the tested inbred lines.

### Evidence of Recombination

The polymorphic sites in the entire *RTCS* sequence were used to detect the evidence of recombination. The patterns of the polymorphisms identified in inbred lines surveyed in this study indicated the history of recombination at *RTCS* gene, which contributed to the haplotype diversity. Under the algorithm of Hudson and Kaplan [21], at least 6 recombination events were found to be responsible to the polymorphism of *RTCS* gene. The recombination events were detected in the informative sites of every region, and they were found in the positions between 5′-UTR and exon-1 (82–353), between exon-1 and intron (398–468), between intron and exon-2 (499–553), the exon-2 (597–667), between exon-2 and 3′-UTR (879–1038), and the 3′UTR (1038–1154), respectively. The consequences of recombination events are evident in the pattern of polymorphisms when compared the sequence of one haplotype with others. For example, the 5′ UTR sequence of the Hap_1 was the same as that of Hap_2. However, across the coding region and intron region, there were 4 variants between them, including 3 SNP and 1 indel covering 3 sites. The 3′UTR region of Hap_1 was found to be again virtually identical to Hap_2. This result suggested that the *RTCS* sequence in Hap_2 has resulted from at least two recombination events in the past relative to Hap_1.

## Discussion

The abundant genetic variations are the foundation for crop improvement. The analysis of the genetic diversity of plant variants is critical for understanding the genetic background of phenotypic variation, and in turn will provide great help for crop improvement [23]. In this study, we detected the nucleotide polymorphisms and the haplotype diversity of the gene *RTCS*, an important regulator for the developing of roots, in 73 China elite maize inbred lines. The identification of nucleotide variations exerting functional effects, especially those causing changes of amino acid composition, is the primary focus of association mapping [13]. Although most variants were found to be located in the non-coding region, the SNP sites and indels in the coding region also classified the tested inbred lines into 14 haplotypes. In addition, a total 7 deferring RTCS proteins were encoded by this gene in all the tested inbred lines. The nucleotide polymorphisms of *RTCS* gene in this study would be helpful in identifying alleles for further genetic analysis, and might also provide foundation for maize improvement.

Heterotic groups are of primary importance in hybrid breeding. Crosses between inbred lines from different heterotic groups generally result in vigorous F1 hybrids with significantly more heterosis than F1 hybrids from inbred lines within the same heterotic group [24]. Heterotic groups are created by plant breeders to classify inbred lines, and can be progressively improved by reciprocal recurrent selection [25]. Although the classification of inbred lines into heterotic groups was based on their general combining ability (GCA) and specific combining ability (SCA) effects, the inbred lines within one heterotic group were generally believed to possess lower genetic divergence than those between different groups. Thus, molecular data, especially SSR molecular markers, was thought to be the efficient method in assigning inbred lines to specific heterotic groups [24], [26], [27], [28], [29]. The nucleotide polymorphisms of the *RTCS* locus were investigated in 73 elite inbred lines from different heterotic groups. The results revealed that sequence variants within each group were higher or very near to those of the whole set except for P group for both the entire region and the coding sequences. Because breeders mainly focused on increasing shoot biomass and seed yield in maize improvement in the past, the relevance of the root system for crop improvement has often overlooked [1], [2]. The abundant variants within one heterotic group might be the result of overlook in the selection by breeders, although this gene plays important roles in formation of seminal and shoot-borne roots.

The purpose of the selection test is to distinguish between a DNA sequence evolving randomly (neutrally) and one evolving under a non-random process, including directional selection or balancing selection, demographic expansion or contraction, genetic hitchhiking, or introgression [19]. The randomly evolving mutations are called “neutral”, while mutations under selection are “non-neutral”. In this study, we performed selective analysis for each heterotic group, and the results revealed that only P group was influenced by strong negative selection. Other groups have not influenced by selection, suggesting that a bottleneck for the usage of this locus in breeding in these heterotic groups. In addition, the haplotype detection also found that P group has a lower value of haplotype diversity than others. This might be the result of that this group was used in China for a short period after 1980s, and most of the inbred lines of this group in China were selected from the pioneer hybrid P78599 [26]. The consistency of the genetic background for the inbred lines in P group resulted in the low frequency of nucleotide variants. Huangzaosi is the most used maize inbred line in China, and more than 42 hybrids and 70 derived lines used this inbred line since it was first bred in 1971 [22]. 11 inbred lines of Huangzaosi and its derived lines were used to test the nucleotide polymorphisms. The results revealed that this set has a higher nucleotide diversity than the whole set, and no selection was identified in this set. These result suggested that the *RTCS* locus was not adopted when the breeders used Huangzaosi as a key inbred line.

## Supporting Information

Table S1 **The positions of nucleotide polymorphism of** ***RTCS*** **gene among 73 inbred lines.**
(XLS)

Table S2 **The distribution of haplotypes of** ***RTCS*** **gene in 73 inbred lines using the entire sequences.**
(DOC)

Table S3 **The distribution of haplotypes of** ***RTCS*** **gene in 73 inbred lines using the coding sequences.**
(DOC)
