# Supplementary material for: Nucleotide Polymorphisms and Haplotype Diversity of RTCS Gene in China Elite Maize Inbred Lines
Source: PLoS One. 2013 Feb 20;8(2):e56495. doi: 10.1371/journal.pone.0056495 (PMC3577901; doi:10.1371/journal.pone.0056495)
Supplement: Table S2 — The distribution of haplotypes of RTCS gene in 73 inbred lines using the entire sequences. (DOC) [file pone.0056495.s002.doc]

**Table S2 The distribution of haplotypes of *RTCS* gene in 73 inbred lines using the entire sequences**

| **Haplotype** | **Number** | **Inbred Lines** |
| --- | --- | --- |
| Hap_1 | 5 | Ji853, DK3110, Luyuan92, BJ-5, B73 |
| Hap_2 | 1 | Y53 |
| Hap_3 | 1 | 10533-1 |
| Hap_4 | 1 | 11099 |
| Hap_5 | 1 | FLB01 |
| Hap_6 | 2 | k12, Zheng58 |
| Hap_7 | 1 | BEM |
| Hap_8 | 21 | Qi318, BJ-3, xy35, Qi319, 10168, JB, Danhuang25, Shen137, S122, Tie9206, Exhan, 6819, QP1721, 319B, Dan988, Chang7-2, P138, 178, 4CV, 340Gai, Dan99 |
| Hap_9 | 2 | 4866, s80 |
| Hap_10 | 1 | BJ-4 |
| Hap_11 | 1 | GB28 |
| Hap_12 | 1 | 11118 |
| Hap_13 | 1 | suwan |
| Hap_14 | 1 | Benyu15 |
| Hap_15 | 1 | LX9801 |
| Hap_16 | 1 | QH19612 |
| Hap_17 | 1 | H21 |
| Hap_18 | 2 | Dan598, RBS11 |
| Hap_19 | 1 | 502 |
| Hap_20 | 3 | 11200, 107, Wu314 |
| Hap_21 | 1 | Mo17 |
| Hap_22 | 1 | 3189 |
| Hap_23 | 1 | Za107 |
| Hap_24 | 3 | OH43, 7922, 8112 |
| Hap_25 | 1 | JH3372 |
| Hap_26 | 1 | K8112 |
| Hap_27 | 9 | E28, QZ01, nx335, Zong3, 91158, BJ-1, Qi232, 412, Huangzaosi |
| Hap_28 | 1 | Huang518 |
| Hap_29 | 1 | Zi330 |
| Hap_30 | 1 | Dan340 |
| Hap_31 | 1 | RCML15 |
| Hap_32 | 1 | 478Xuan |
| Hap_33 | 1 | 8605-2 |
| Hap_34 | 1 | Chun2433 |
