# Supplementary material for: Nucleotide Polymorphisms and Haplotype Diversity of RTCS Gene in China Elite Maize Inbred Lines
Source: PLoS One. 2013 Feb 20;8(2):e56495. doi: 10.1371/journal.pone.0056495 (PMC3577901; doi:10.1371/journal.pone.0056495)
Supplement: Table S3 — The distribution of haplotypes of RTCS gene in 73 inbred lines using the coding sequences. (DOC) [file pone.0056495.s003.doc]

Table S3 The distribution of haplotypes of *RTCS* gene in 73 inbred lines using the coding sequences

| CDS haplotype | Haplotypes | Number of inbred lines |
| --- | --- | --- |
| CDS_Hap_1 | Hap_1, Hap_3 | 6 |
| CDS_Hap_2 | Hap_2 | 1 |
| CDS_Hap_3 | Hap_4 | 1 |
| CDS_Hap_4 | Hap_5 | 1 |
| CDS_Hap_5 | Hap_6, Hap_7, Hap_8, Hap_9, Hap_10, Hap_11, Hap_12 | 29 |
| CDS_Hap_6 | Hap_13, Hap_17 | 2 |
| CDS_Hap_7 | Hap_14, Hap_15, Hap_16, Hap_18, Hap_19, Hap_20, Hap_21 | 10 |
| CDS_Hap_8 | Hap_22, Hap_23, Hap_24, Hap_25, Hap_27, Hap_28, Hap_29 | 17 |
| CDS_Hap_9 | Hap_26 | 1 |
| CDS_Hap_10 | Hap_30 | 1 |
| CDS_Hap_11 | Hap_31 | 1 |
| CDS_Hap_12 | Hap_32 | 1 |
| CDS_Hap_13 | Hap_33 | 1 |
| CDS_Hap_14 | Hap_34 | 1 |
